# Developing new lines of Japonica rice for higher quality and yield under arid conditions

**DOI:** 10.7717/peerj.11592

**Published:** 2021-06-14

**Authors:** Mahmoud A.A. El Sayed, Ahmed M.S. Kheir, Fatma A. Hussein, Esmat F. Ali, Mahmoud E. Selim, Ali Majrashi, Essam A.Z. El Shamey

**Affiliations:** 1 Rice Research Department, Field Crops Research Institute, Agricultural Research Center, Egypt; 2 Soils, Water and Environment Research Institute, Agriculture Research Centre, Egypt; 3 Department of Biology, College of Science, Taif University, Taif, Saudi Arabia

**Keywords:** Recent cultivars, Breeding, Genetics, Genotypes, Phenotypes, Hybridization

## Abstract

Rice is the world’s largest food crop, and its production needs to be doubled by 2050 to cope with population growth and associated demand. In addition to the value of improving yields, quality is also important for breeders and consumers, but it pays less attention in arid regions. During two successive summer growing seasons, the experimental material focused on 34 genotypes developed from different crosses on Fn generation after fixation as well as six of the most recent commercial cultivars used for comparisons. The results showed that a high yield of grain followed by high milling and grain quality characteristics were observed among the 34 genotypes used in this analysis. Highly important and positive correlations between the percentage of hulling and the percentage of milling (0.424) and the yield ability could be accomplished by choosing the number of panicles per plant and the weight of the panicles. Selection criteria for good quality should be met by the percentage of head rice and many mineral elements, particularly zinc and iron. As a consequence, the genotypes M.J 5460S/SK105-1, M.J 5460S/GZ7768-1, M.J 5460S/G177-1, M.J 5460S/SK105-3 and M.J 5460S/SK106-4 had desirable high yield and quality characteristics and could be used as promising accessions to the rice breeding program in arid regions. In addition to commercial genotypes, improved Japonica rice genotypes could be produced in arid conditions for higher yield and quality, leading to an increase in total production, supporting food security and nutrition.

## Introduction

Rice (*Oryza sativa* L.) is one of the world’s most important food crops, feeding more than half of the world’s population (IRRI, 2010; Baroudy et al., 2020). It is also one of the most valuable crops and the world’s second-largest cereal crop, after wheat, with 430 million metric tons produced (IRRI, 2009). However, the sustainable production of rice facing different constraints including the competition of resources, soil, water, climate change, labor availability, and the cost of corresponding production. Other critical factors influencing sustainable crop production in arid and semi-arid regions include abiotic stress (Kheir et al., 2021), soil quality (Shokr et al., 2021),and limited water resources (Ding et al., 2021). Previous research demonstrated the significance of newly used cultivars (Gomaa et al., 2015; Selim et al., 2020), but comparisons between such genotypes and japonica lines have received less attention thus far. An appropriate breeding program is therefore necessary to boost productivity in order to meet the future demand associated with over population growth (Godfray et al., 2010). In addition to improving yield, quality is another key that challenge needs to be considered in breeding programs (Usman et al., 2020). Grain quality is usually categorized in rice into four components: milling ability, appearance, characteristics of cooking and eating and nutritional value (Peng et al., 2014). Brown rice percentage (BRP), milled rice percentage (MRP) and head rice percentage (HRP) are usually evaluated as milling quality (Bodie et al., 2019). The gelatinization temperature (GT), amylose content (AC), and gel intensity (GC) of the grain endosperm specify the cooking and eating characteristics (Wang et al., 2019). Among these, characteristics of milling and appearance are closely correlated with grain shape. In general, grain milling efficiency is negatively correlated with rice length (RL), rice length to width ratio (RLWR), or length to thickness ratio, whereas increased rice width (RW) and thickness tend to result in increased milling efficiency (Jongkaewwattana & Geng, 2020). Rice grain quality choice varies from country to country and from region to region. Zinc and iron deficiency (Zhao et al., 2020a) increases the risk and severity of common childhood infections, impacting babies’ survival, and leads to stunting in young children’s development (Brown et al., 2004). Latest estimates indicate that about four percent of infant mortality and disability-adjusted life-years worldwide could be responsible for zinc and iron deficiency (Black et al., 2008). In addition, such elements are very critical to support the system of human immunity in the fight against pandemic diseases such as Covid-19. To avoid Zn and Fe deficiency in rice as one of the most reliable food crops, measures are urgently needed for these reasons. In Egypt, as an important exporting rice country, various higher yielded rice cultivars have recently been introduced to commercial cultivars such as Giza177, Giza178, Giza179, Sakha super300, Sakha101 and Sakha108. There are substantial differences between Japonica and Indica rice varieties (Liu & Li, 2016). Major differences might be noticed in leaf photosynthesis per area, photosynthesis *N* use efficiency, and specific leaf weight (Liu & Li, 2016). In addition, the grain in Japonica is short and round, yet Indica is slender. Furthermore, the quality of rice milled, the degree of chalkiness (C) and the consistency of gel (GC) of Japonica is more desired than that of Indica, its amylose (AC), gelatinization (GT) and protein (PC) content is lower than that of Indica rice (Olsen et al., 2006). Other researchers found that the interactions between the characteristics of subspecies and ecosystem environments had some effects on the appearance and milled rice quality (Mao et al., 2010; Wei et al., 2020). However, the comparison between Japanese and Indica genotypes in arid and semi-arid conditions is still uncertain, requiring further comparison. The present study was aimed at estimating some grain quality characteristics of new Japonica genotypes compared to some Egyptian commercial varieties with higher grain yield and quality characteristics.

## Materials and Methods

### Experimental location and hybridization

The experimental research work of this investigation was conducted at of the research farm in Sakha Agriculture Research Station, Rice Research Department, Sakha, Kafr El Sheikh, Egypt, during two successive summer seasons 2019 and 2020. The plant materials consisted of 34 genotypes of the Fn generation after stability of four crosses, in addition to six commercial Egyptian varieties of rice; Giza178, Giza177, Giza179, and Sakha Super300, as well as Sakha101 and Sakha108, which were the best two commercial varieties grown under Egyptian conditions and used for comparison. The crosses were made by crossing one reverse-thermo responsive Genic Male Sterile (rTGMS) line, M.J 5460S, with three Egyptian cultivars, Giza177, Sakha105, Sakha106, and one promising line, GZ.7768, as male lines in cross I (10 Fn genotypes), cross II (3 Fn genotypes), cross III (11 Fn genotypes), and cross IV (10 Fn genotypes), respectively. Table S1 shows rice parental lines, parentage origin, and grain form. Pedigree selection methods in segregation generation from F2 to F6 were used after hybridization to ensure stability. In experimental plots, 30-day-old seedlings of each genotype were transplanted into one seedling per hill. Each plot consisted of seven rows with a length of 5 m and a spacing of 20 × 20 cm in three replications. Cultural practices were applied as recommended by Recommendations of Rice Research and Training Center (RRTC). The studied characters were days to heading (day), plant height (cm), number of panicle plant^−1^, panicle weight (g), panicle length (cm), yield m^−2^ (kg), total grain panicle^−1^ and harvest index (%). Days to heading were determined as number of days from date of sowing to the date of the first panicle exertion of each plant. Plant height is the length of the main culm measured from soil surface to the tip of the panicle. Panicle’s plant^−1^ was counted when all panicles were at the full ripe stage. Panicle weight was recorded by weighting the main panicle in the plant after drying moisture. Panicle length is the main panicle was measured from panicle base up to the upper most spikelet of the panicle. Grains panicles^−1^ was recorded as the total number of grains in one panicle. Grain yield plant^−1^ was recorded as the weight of grain yield of each individual plant. Harvest index was calculated by divided grain yield by biological yield.

### Climate, soil and water status

**Figure 1:**
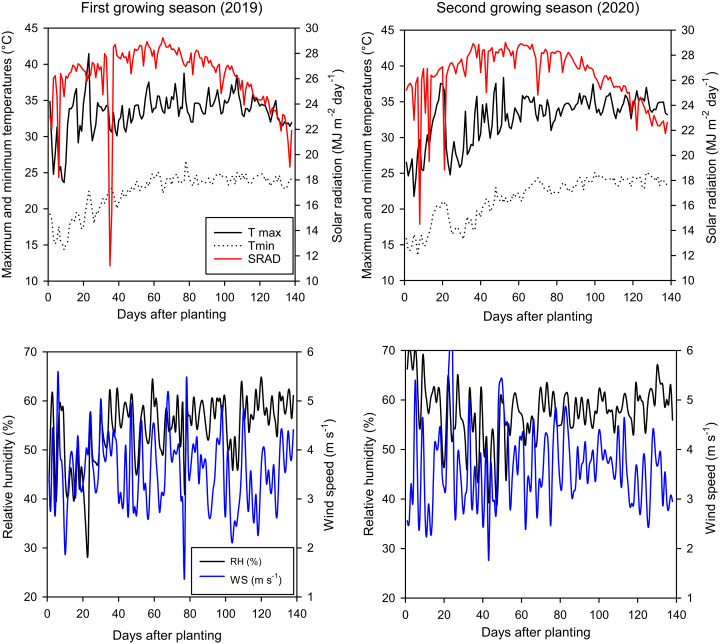
Daily climatic data of maximum and minimum temperatures (Tmax and Tmin), solar radiation (SRAD), relative humidity (RH), as well as wind speed (WS) in the studied area over two growing seasons (2019 and 2020). Measured climatic data were collected from an automated weather station close to the field experiment.

The study area has a hot climate in the summer, with no rainfall and higher temperatures (Fig. 1). The maximum temperature range is 23–42 °C, the minimum temperature is 12–19 °C, the solar radiation is 111-29 MJ day^−1^ m^−2^, the relative humidity is 40–65%, and the wind speed is 1.8–6 m s^−1^. Interestingly, maximum and minimum temperatures were higher in first growing season than second season, while relative humidity and wind speed were higher in second growing season than first season. The soil in the study area is a clay old Nile valley soil (18.5% sand, 22.5% silt, and 59% clay). It also has a higher water holding capacity due to its higher moisture content at field capacity (42%), available water (22%), and lower bulk density (1.2 g cm^−3^).

### Statistical analysis

The analysis of variance for each trait was done using the Statistic Analysis System (SAS) version 9.2 for windows. Yield and its components and grain quality were used to estimate the genotypic variance (σ2g), environmental variance (σ2e) and phenotypic variance (σ2p) as described by Adjah et al. (2020). The σ2g and σ2e were tested using the standard error. The variance components were used to compute the genotypic coefficient of variation (GCV), error coefficient of variation (ECV), the phenotypic coefficient of variation (PCV) as outlined by Falconer (1996), broad-sense heritability (H2) according to Allard (1999). Principal Component Analysis (PCA) for yield and quality parameters of all genotypes was performed using Prcomp and HCPC functions and FactoMineR and FactoExtra packages in RStudio.

### Quality categories

Duplicate 150 g of rough rice from each variety were used for hulling percentage determination and calculated according to method mentioned by Yao et al. (2017) through dividing the brown rice weight by total rough rice weight. The objective of the rice milling is to remove the bran and germ with the minimum endosperm breakage. It was also determined on the basis of Bodie et al. (2019). The whole grains (head rice) were separated according to the broken size (less than 1/4th of grain length) with rice-sizing device and then weighted. Head rice percentage was determined through dividing weight of head rice to rough weight. Grain size (length and width) was taken from 50 normal grains of each plot using a Micrometer. The length/width ratio (grain shape) was calculated from these values and the following scale as suggested by Yao et al. (2017) was described the grain shape through length/width ratio as follow; slender (>3.0); medium (2.1 to 3.0); bold (1.1 to 2.0) and round (<1.0). The length of cooked grains was measured in millimeters. Average length of row and cooked grains was calculated. The proportionate change (PC) in L/W ratio was calculated according (Sood & Siddiq, 1980). Such alkali spreading and clearing of starchy endosperm represented the GT which was visually rated on seven–point numerical scale adopted by Little, Hilder & Dowson (1958). Amylose content % was determined according to the methods of Williams et al. (1958) as follow: Amylose content was determined by reference to a standard curve and expressed on a dry weight basis. Calculation of the conversion factor was performed by plotting the absorbance values at (620 mu) against the concentration of anhydrous amylase (mg). Samples were then diluted by 20 for the conversion process. To prepare samples for Fe and Zn analysis, they are dried to a moisture content of 12–14% in the air. A Palm de husker made from rubber was used for hulling. The lemma and palea were extracted during hand hulling in order to prepare fine power with a pestle and mortar. A 500 mg of powdered samples was taken in a 100 ml conical flask. The sample was treated with 12 ml of a triple acid mixture (9:2:1 Nitric, Sulfuric, and Perchloric acid) and kept cold for overnight digestion. Meanwhile, the digested samples were kept on a hot plate until reaching colorless. Then the extract was diluted to 50 ml and fed to the Atomic Absorption Spectrophotometer (GBC Avanta version 2.02), the readings were expressed as mg/kg.

## Results

The development of new genotypes technology and adoption of cultivars to Egyptian environment offer an approach to the problem of matching food supply with expected demand. Forty genotypes were chosen and used to study some yield and its components characters and quality grain traits. To better represent and discuss the results achieved, it was preferred to outline these results under two main topics. The first issue concerns yield genetics and its components, as well as quality grain characteristics, while the second is the genetic diversity of brown rice with iron and zinc content.

### Mean performance of yield, its components and quality

The data summarized in Table S2 show that the mean values of the genotypes were (98.4-day, 101.3 cm, 21.6, 5.5 g, 21.4 cm, 208.8, 1.3 kg, and 43.5%) for days to heading, plant height, number of panicles per plant, panicle weight, panicle length, total grains per panicle, yield per m^2^, and harvest index %, respectively. For days to heading, the most desirable mean values for earliness were derived from the lines; M.J 5460S/G177-1 (89.6 day) and M.J 5460S/G177-4 (90.3 day) when compared with the best earliness of the commercial varieties Giza177 (92 day). The results also revealed that, the most desirable mean values towards dwarf were found for the line M.J 5460S/Sk106-3 since this gave the lowest value for plant height. On the contrary, the highest value was recorded with the line M.J 5460S/G177-10. For the number of panicles per plant character, the highest mean values were obtained by the lines; M.J 5460S/SK105-2, M.J 5460S/Sk106-5 and M.J 5460S/Sk106-10 (30.0, 27.3 and 27.3 panicles/plant), respectively and if it compared with the best commercial variety has highest number of panicle per plant (Giza179 and Sakha108) which gave mean values of 24.7. The desirable mean values for panicle weight were recorded by the two lines; M.J 5460S/G177-3 and M.J 5460S/Sk106-10 with values of (7.5 and 7.0 g), respectively and lowest value was recorded with the line M.J 5460S/G177-6 which gave 3.5 g, since this gave the lowest value for panicle weight Sakha108 (3.6 g). Meanwhile, for panicle length, the lines M.J 5460S/GZ7768-4 and M.J 5460S/GZ7768-5 gave the desirable mean values (24.3 and 24.6 cm), respectively in Table 1, and the best commercial variety Sakha101 gave the mean value (24.7 cm). Regarding the total grains per panicle character, the desirable mean values were recorded for the lines M.J 5460S/GZ7768-10, M.J 5460S/GZ7768-4, and M.J 5460S/G177-3 which gave (297. 7, 266. 7, and 264. 7 grain/panicle), respectively with respect to the best commercial variety in these character Sakha Super300 which gave (235.00 grain/panicle). For yield per m^2^ character, the yield productivity ranged between (1.51 and 1.03 kg) with the lines M.J 5460S/GZ7768-1 and M.J 5460S/SK106-11, respectively and the best commercial variety gave 1.24 kg with Sakha Super300 which recorded the highest mean values for this character. Also, it could be noticed that the desirable mean values for harvest index % character were recorded for the two lines; M.J 5460S/GZ7768-3 and M.J 5460S/g177-10 which gave (49.90 and 49.9%), respectively and the commercial variety Giza178 gave the best value among commercial verities which used at this study (48.6%). These genotypes may be considered as ideal sources for varieties improvement through pedigree breeding or bi-parental meeting.

**Table 1:** Mean square analysis for yield and its component and grain quality characters.

Characters	Rep (df = 2)	Genotypes (df = 39)	LSD	CV
Days to heading	1.929 ns	72.64**	3.36	4.53
Plant height	6.408 ns	602.59**	4.51	7.92
Panicle plant^−1^	2.508 ns	44.816**	3.57	23.31
Panicle weight	0.162 ns	3.216**	0.17	0.214
Panicle length	0.418 ns	7.752**	0.81	1.196
Total grain panicle ^−1^	49.308 ns	3,125.931**	6.42	7.8
Yield m^−2^	0.002 ns	0.062**	0.14	0.64
Harvest index (%)	6.494 ns	53.123**	1.01	0.925
Hulling (%)	0.589 ns	16.553**	1.34	0.87
Milling (%)	0.428 ns	24.391**	0.67	0.24
Head Rice (%)	0.931 ns	66.128**	1.01	0.61
Amylose (%)	0.279 ns	2.53**	0.21	0.1
GT	0.071 ns	1.91**	0.27	0.47
Elongation	0.983 ns	212.53**	3.68	18.59
Paddy grain shape	0.001 ns	0.064**	0.14	0.32
White grain shape	0.002 ns	0.071**	0.16	0.518

**Notes:**

ns not significant.

* Significant at the 0.05 level.

** High significant at the 0.01 level.

The data in Table S3 indicated that the genotype M.J 5460S/G177-10 was the highest genotype in hulling % (88.8%) followed by genotypes; M.J 5460S/G177-5, M.J 5460S/G177-6, M.J 5460S/G177-7, M.J 5460S/Sk106-4, M.J 5460S/GZ7768-2, and M.J 5460S/GZ7768-5, these genotypes gave the same hulling % (83.7%), while the genotype M.J 5460S/Sk106-7 gave the lowest value (74.1%). But in milling % six genotypes M.J 5460S/G177-9, M.J 5460S/SK105-1, M.J 5460S/SK106-2, M.J 5460S/SK106-3, M.J 5460S/SK106-4, and M.J 5460S/GZ7768-4, recorded the highest milling % value (74.8%).The genotypes M.J 5460S/GZ7768-7 gave the lowest milling % (60.2%). In head rice %, the genotypes M.J 5460S/SK106-11, M.J 5460S/GZ7768-4 and M.J 5460S/SK105-1 gave the highest value (71.3 and 71.2% respectively) and it means that this genotype has the lowest broken grains. For amylose content, the genotypes M.J 5460S/SK106-11 and M.J 5460S/GZ7768-6 gave 20.9% (high amylose content), on the other hand, the genotypes M.J 5460S/G177-8, Sakha101 and M.J 5460S/G177-1 have the lowest amylose content (17.1, 18.0 and 18.00), respectively. For gelatinization temperature (GT), two genotypes M.J5460S/SK105-1 and M.J 5460S/GZ7768-8 gave 7.00 which mean that Kernel completely dispersed and intermingled that’s considered (very low). But the genotype M.J 5460S/SK105-2 recorded 4.00 as the lowest value in GT, which also mean that Kernel swollen, collar complete and wide that is considered (intermediate). In elongation character, the genotypes M.J 5460S/G177-4 and M.J 5460S/GZ7768-8 showed the highest values (41.0−40.0) among all tested genotypes which classifying as medium, but among the Egyptian commercial varieties; the variety Sakha Super 300 gave the highest value (44.5), while the genotype M.J 5460S/GZ7768-4 gave 14.2 as lowest value and it classifying as low in diagram of kernel elongation. For grain shape, the genotypes M.J 5460S/GZ7768-9 and M.J 5460S/Sk106-5 gave the highest values (2.8 and 2.7 respectively), but the genotypes Sakha Super300 and M.J 5460S/GZ7768-5 recorded the lowest values (2.1 and 2.2) respectively in paddy rice grains. But in milling grains, the genotype M.J 5460S/GZ7768-10 recorded the highest value 2.4 followed by M.J 5460S/Sk106-6 and M.J 5460S/GZ7768-2 which gave 2.2 for each one, while the genotype Sakha101 gave the lowest value (1.7) followed by Sakha Super300 and M.J 5460S/G177-8 which gave 1.72, 1.74 respectively. From these data all these genotypes are bold to medium grains. The analysis of variance exhibited highly significant differences among the 40 genotypes (Table 1). It was clear that there were very significant differences between the genotypes for all the characters studied. On the contrary, there were insignificant differences between reps for all yields and their component characters and grain quality characteristics. As a result, the study of all sources of variation revealed that these characteristics have genetic variability within genotypes and that there is no environmental impact. The pedigree method can be used to strengthen all characters under analysis and create new verities, according to the findings summarised above. The number of panicles per plant character had the highest CV (23.31), while amylose % trait had the lowest CV (0.1). The CV values express the experimental error as a percentage of the mean. Therefore, the higher CV value does not mean the reliability of the experiment, but the lower value means that the reliability of the experiment yield per area showed lower CV values compared to the number of panicles per plant with high CV values, indicating that the recurrent selection should be referred to the genetic effect.

It could be noted that the dominant genetic variance as a portion of the total genetic variance was greater than the additive genetic variance for all yields and their component characteristics (Table 2). As far as heritability estimates are concerned, it shows that high values have been determined in a broad sense for all yields and their component characteristics. For panicle weight as an example for yield and its component characters, the data in Table S4 showed that the dominance genetic variance as a portion of the total genetic variance was larger than the additive genetic variance. These results showed that the two components of genetic variance could be important in the inheritance of the length of the panicle, while the predominance of genetic variance played a more important role in this case. As far as heritability estimates are concerned, high value (94.25%) was determined in a broad sense. As shown in Table 2, the heritability is an important concept in quantitative genetics; the table for heritability showed that head rice %, total grains per panicle and plant height have high heritability values, while white grain shape has the lowest value.

**Table 2:** Estimate of genetic parameters, heritability in broad sense, genetic advance for yield and its component and grain quality characters.

Characters	Mean	Range		σ^2^_g_	σ^2^_e_	σ^2^_P_	GCV (%)	ECV (%)	PCV (%)	H^2^ (%)
		Min	Max							
Days to heading	98.41	89.62	109.03	22.73	6.39	29.12	4.84	2.57	5.48	78.07
Plant height	101.34	77	124	198.19	14.44	212.62	13.89	3.75	14.39	93.21
Panicle plant^−1^	21.58	15.67	30	13.26	7.54	20.8	16.87	12.72	21.13	63.75
Panicle weight	5.45	3.48	7.52	1.07	0.174	1.24	18.98	7.65	20.46	86.01
Panicle length	21.42	17.75	24.67	2.5	0.67	3.17	7.38	3.83	8.31	78.76
Total grain panicle^−1^	208.84	144.67	297.67	1,036.55	65.6	1,102.15	15.42	3.88	15.9	94.05
Yield m^−2^	1.27	1.03	1.51	0.02	0.01	0.03	10.55	8	13.24	63.49
Harvest index (%)	43.46	34.66	49.9	17.57	6.9	24.47	9.65	6.04	11.38	71.82
Hulling (%)	81.28	74.11	88.76	5.28	1.29	6.58	2.83	1.4	3.16	80.33
Milling (%)	71.96	60.16	74.83	8.07	0.604	8.68	3.95	1.08	4.09	93.04
Head Rice (%)	66.44	51.55	72.64	21.91	1.34	23.24	7.05	1.74	7.26	94.25
Amylose (%)	19.19	17.08	20.91	0.836	0.296	1.13	4.76	2.84	5.55	73.83
GT	5.98	4	7.01	0.625	0.1	0.725	13.21	5.27	14.22	86.26
Elongation	28.8	14.15	44.52	69.06	6.34	75.4	28.85	8.74	30.15	91.59
Paddy grain shape	2.35	2.11	2.78	0.02	0.01	0.03	5.83	4	7.07	68
White grain shape	1.98	1.71	2.23	0.02	0.012	0.032	7.22	5.49	9.07	63.34

**Note:**

σ^2^g Genotype variance; σ^2^e Environmental variance; σ^2^P Phenotype variance; GCV Genotype coefficient of variation; ECV Error coefficient of variation; PCV Phenotype coefficient of variation; H2 Broad sense heritability; GA Genetic advance as percent of mean.

The results in Table S4 revealed that highly significant and positive correlations among hulling % and milling % (0.424), and total grain per panicle with harvest index % (0.513), and yield per m^2^ with harvest index % (0.462). The correlation co-efficient between yield m^2^ with number of panicles per plant was highly significant and positive and recorded value (0.814). In correlation coefficient analysis, brown rice recovery, milling recovery and head rice recovery had significant positive association among themselves. Meanwhile, GT character showed a significant correlation with hulling %, milling and head rice %. But for amylose content there is a negative correlation with GT and hulling % while negative and significant correlation with milling %. Also, there is a positive correlation and significance between elongation and other quality characters under this study for gelatinization temperature (0.279). Also, there are negative and highly significant correlation between paddy grain shape and both head rice % (−0.300), and elongation (−0.360) in addition white grain shape and kernel elongation there is negative and highly significant correlation with (−0.390).

### Genetic diversity of brown rice for iron and zinc content

Both iron and zinc are known as microelements that play an important role within the plant, whether in oxidation and reduction reactions or respiration and other vital processes in the plant, due to the wrong fertilization and the excess of some elements beyond the permissible limit, which led to the difficulty of absorbing both iron and zinc by the plant and we solve this problem by using some compounds such as potassium to facilitate iron in the soil and zinc sulfate to increase the proportion of zinc in the soil as well. Therefore, we find that the presence of some new lines that contain high levels of iron and zinc, which will have an important role, as the consumer can use them to help in the recovery of anemia and some immune diseases that affect children and women, especially in poor areas. Data in Table 3 showed that the genotypes (M.J 5460S/G177-1, M.J 5460S/SK105-1and M.J 5460S/SK105-2) recorded the highest value in Iron (Fe) concentration which gave (27.50, 27.50 and 26.00 mg/kg respectively), while the genotype (M.J 5460S/G177-9) gave the lowest value. On the contrary, the genotypes (M.J 5460S/SK105-1 and M.J 5460S/GZ7768-8) gave the highest values of Zinc concentration Zn (133.75 and 127 mg/kg respectively. But the genotype (M.J 5460S/G177-1) gave the lowest value (12.65 mg/kg).

**Table 3:** Minerals content in Brown rice for some selected rice genotypes.

Genotypes	Elements (mg kg^−1^)	
	Fe	Zn
M.J 5460S/G177-1	27.5 ± 1.7	12.65 ± 1.1
M.J 5460S/G177-9	10.5 ± 1.1	14.65 ± 1.4
M.J 5460S/G177-4	17.25 ± 1.5	23.65 ± 1.5
M.J 5460S/SK105-1	27.5 ± 1.9	133.75 ± 3.5
M.J 5460S/SK105-2	26 ± 2.5	24.17 ± 1.2
M.J 5460S/SK105-3	20.5 ± 2.2	16.35 ± 0.5
M.J 5460S/SK106-4	16.5 ± 1.4	14.9 ± 0.6
M.J 5460S/SK106-6	11.5 ± 0.8	14.3 ± 0.7
M.J 5460S/SK106-10	11.75 ± 0.7	16.95 ± 0.8
M.J 5460S/GZ7768-1	19.5 ± 0.6	36.57 ± 2.1
M.J 5460S/GZ7768-6	21 ± 0.5	14.17 ± 1.3
M.J 5460S/GZ7768-8	23.75 ± 1.1	127 ± 3.8
Giza 177	11.5 ± 1.5	15.3 ± 0.5
Sakha 108	17.5 ± 0.7	18.35 ± 1.1
Sakha 106	12.5 ± 0.6	15.77 ± 0.5

**Note:**

Fe: iron, Zn: zinc.

## Discussion

**Figure 2:**
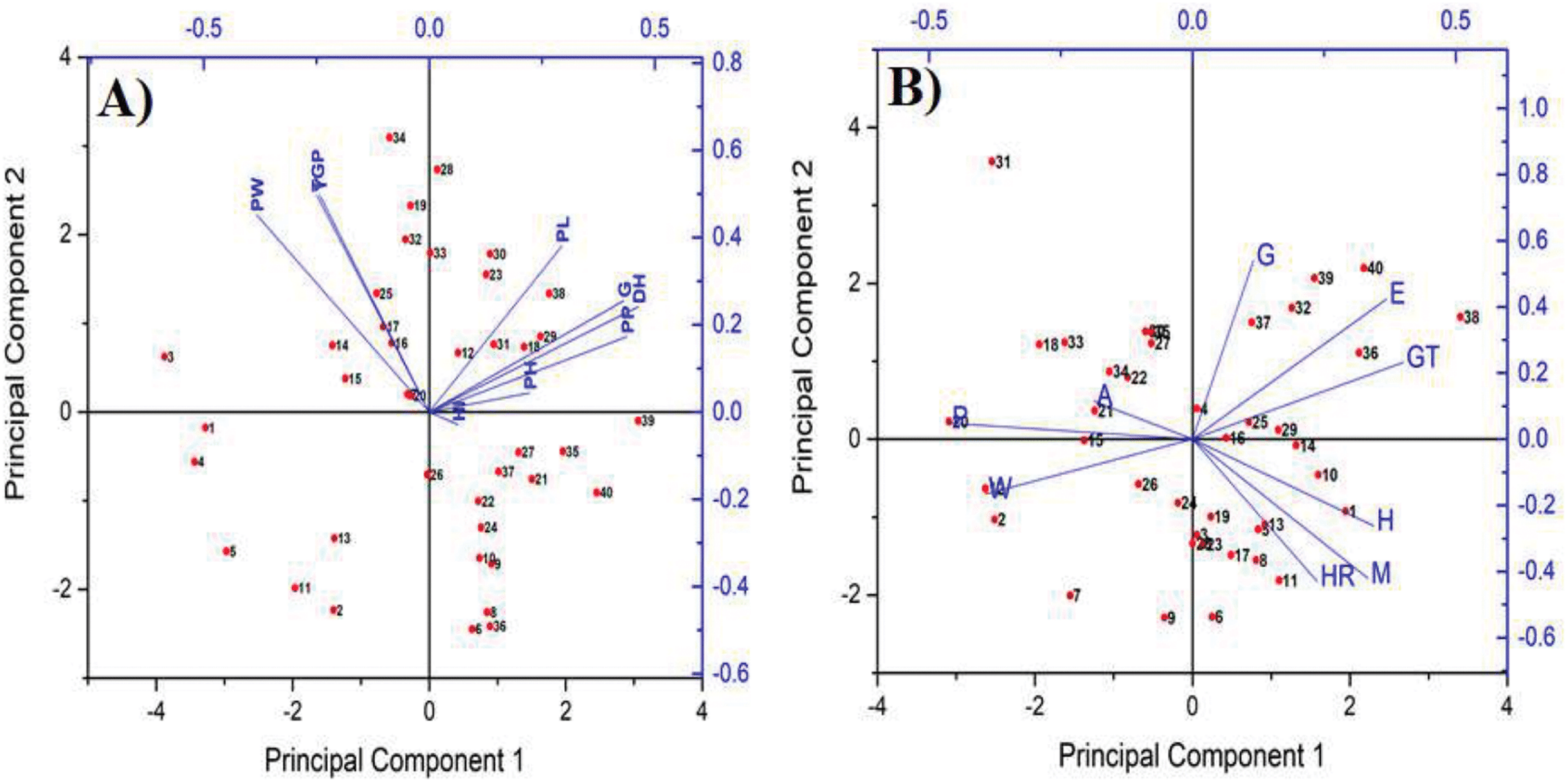
Principle component analysis (PCA) to better understand the variability of genotypes (red dots) and yield (A) with its components (blue vectors) as well as quality (B) (blue vectors). In (A) yied and its attributes are DH, PH, PP, PW, PL, TGP, Y and HI for days to heading, plant height, panicle plant^−1^, panicle weight, panicle length, total grain panicle^−1^, yield per m^−2^ and harvest index, respectively. Meanwhile in (B) the quality parameters include H, M, HR, A, GT, E, P, and W for hulling, milling, head rice, amylose, gelatinization temperature, elongation, paddy grain shape and white grain shape respectively. The genotypes from 1 at 40 are M.J 5460S/G177-1, M.J 5460S/G177-2, M.J 5460S/G177-3, M.J 5460S/G177-4, M.J 5460S/G177-5,M.J 5460S/G177-6, M.J 5460S/G177-7, M.J 5460S/G177-8, M.J 5460S/G177-9, M.J 5460S/G177-10 M.J 5460S/SK105-1, M.J 5460S/SK105-2, M.J 5460S/SK105-3, M.J 5460S/Sk106-1, M.J 5460S/Sk106-2, M.J 5460S/Sk106-3, M.J 5460S/Sk106-4, M.J 5460S/Sk106-5, M.J 5460S/Sk106-6, M.J 5460S/Sk106-7, M.J 5460S/Sk106-8, M.J 5460S/Sk106-9, M.J 5460S/Sk106-10, M.J 5460S/SK106-11, M.J 5460S/GZ7768-1,M.J 5460S/GZ7768-2, M.J 5460S/GZ7768-3, M.J 5460S/GZ7768-4, M.J 5460S/GZ7768-5, M.J 5460S/GZ7768-6, M.J 5460S/GZ7768-7, M.J 5460S/GZ7768-8, M.J 5460S/GZ7768-9, M.J 5460S/GZ7768-10, Giza178 (CK), Giza177 (CK), Giza179 (CK), Sakha Super300 (CK), Sakha101 (CK), Sakha108 (CK) respectively.

Mean performance of yield and its attributes for the studied genotypes showed higher yield with some lines. For high yield potential hybrids, particularly interspecific hybrids, selecting parental lines with appropriate plant height and non–loading characters is critical (Kumar et al., 2020). These findings are similar to those obtained by Li et al. (2004) who found that, Zhen feng accessions showed good plant type, high combining ability, good flowering habits, high harvest index rate and high yield of seeds. The principle components of a collection of points in a real *p*-space are a sequence of *p* direction vectors, where the yield and its components characters vector are the direction of a line that best fits the gain data from genotypes under this study (Fig. 2A). It was clear that there is a positive relationship between some genotypes and the yield and the characteristics of its components. For example, some genotypes have given desirable productivity yield values such as M.J 5460S/Sk106-6, M.J 5460S/GZ7768-1, M.J 5460S/GZ7768-8 compared to the best Egyptian commercial varieties Sakha super 300. Also, some yield characters related each other like, grain yield/m^2^ with panicle weight, grain panicle^−1^ and panicle plant^−1^, as well as, harvest index with both panicle plant^−1^ and plant height. The principle component 1 and principle component 2 present most characters related with yield productivity under the same group with genotypes had high yield productivity at PCA points (0.5 and 0.2), on the other hand most genotypes were earliness under the same group with genotypes had lowest day to maturity like M.J 5460S/G177-1 and M.J 5460S/G177-4 if it compared with the best earliness commercial varieties Giza 177 at PCA points (2.0 and 0.2). Next to increasing yields, the most important goal is to improve grain quality in order to satisfy customer needs and business demands (Okpiaifo et al., 2020). As a result, rice breeders all over the world are focusing their efforts on developing rice varieties with attractive milling and appearance qualities (Wang et al., 2017). The quality desired in rice varies from one geographical region to another, and consumers are demanding certain varieties and favoring the special quality characteristics of milled rice for home cooking (Custodio et al., 2019). Long grain rice with an intermediate gelatinization temperature is favored in Indica rice-consuming countries because it becomes soft and fluffy after cooking (Pang et al., 2016). Physicochemical characteristics include kernel length, kernel width, kernel shape; and cooking quality includes alkali digestion value, gelatinization temperature and elongation ratio are important for assessing the quality and performance of rice from one consumer group to another (Zohoun et al., 2018). Principle Component Analysis (PCA) is the process of computing the main components and using them to change the basis of the data obtained from the quality characteristics of the genotypes used (Fig. 2B). This figure presents the principle component 1 and principle component 2 and their relationship to quality traits and genotypes under current study, showing a positive relationship between genotypes and quality traits. For example, there are some genotypes gave desirable values to quality characters as M.J 5460S/SK105-1, M.J 5460S/GZ7768-4, and M.J 5460S/SK106-11 if compared with the best Egyptian commercial varieties Giza177 and Sakha super300. Also, some quality traits related each other like, hulling milling. Milling efficiency, appearance, cooking and eating characteristics, and nutritional value are the four components of grain quality in rice. From these components, milling and appearance traits are highly correlated with grain shape. The preference of rice quality varies from region to another worldwide. Low amylose and short grain rice, for example, is preferred in Japonica rice-eating countries because it is soft and sticky after cooking. Meanwhile, long grain rice with intermediate amylose and intermediate gelatinization temperatures is favored in Indica rice-consuming countries because it is soft and fluffy after frying. PCA is a dimensionality reduction technique that involves projecting each data point onto only the first few principal components in order to obtain lower dimensional data while maintaining as much data variance as possible. In addition to early maturity, rich in Fe and Zn nutritional elements, we can summarize our goals to produce Japanese rice with good quality characteristics and high yield productivity. The coefficient of variation was higher for number of panicles per plant and lower with amylose. Similar results reported higher coefficients of variation for brown rice recovery and milled rice recovery (Zhang et al., 2020). The lower CV observed for most yield and quality characteristics in our study may be due to the use of different germplasms. Regarding the estimation of the genetic components and heritability in broad senses, the two components of genetic variance could be important in the inheritance of the length of the panicle, while the predominance of genetic variance played a more important role in this case. As far as heritability estimates are concerned, higher value was determined in a broad sense. These findings are consistent with those obtained earlier from the breakdown of genetic variance in this study. These results showed that this character was influenced by the environmental impact, meaning that the selection for panicle weight might be practiced successfully in late generations. Similar findings have been noted by Kulkarni et al. (2020) and Zhi-yuan et al. (2015). The CV values indicate the degree of precision with which the treatments are compared and are a good index for the reliability of the experiment. The yield capability could be achieved by selecting the number of panicles per plant and the weight of the panicle characters. This may be due to the fact that the yield potential was mainly attributed to the number of grains per panicle and panicle weight (Zhao et al., 2020b). All these components caused heavy weight of panicles and high yielding ability as well as a greater number of panicles plant^−1^. This suggests that, yield can be improved through simultaneous component improvement using suitable selection indices (Michel et al., 2019). The variability in nutritional content among genotypes could confirm that, brown genotypes include higher Zn content than white genotypes (Islam et al., 2013; Majumder, Datta & Datta, 2019). Minerals are well-known basic nutrients that play an important role in the body’s proper functioning (Wang et al., 2017). In comparison to other micronutrients, Fe and Zn are two of the most essential for the human body. In rice, the genetic characteristics of accessions and environmental factors are responsible for affecting mineral content (Verma & Srivastav, 2017). In addition, genotypic variations may provide opportunities to select higher mineral-element rice germplasm (Pinson et al., 2015). Consequently, determining the natural genetic variability of the elements is critical, since an increase in mineral content will contribute significantly to the recommended dietary allowance.

## Conclusions

It is well known that Japonica rice varieties had high quality, so our investigation some genotypes have improved, such as M.J 5460S/SK105-1, M.J 5460S/GZ7768-1, M.J 5460S/G177-1 and M.J 5460S/SK105-3, with high yield and good quality. Owing to their various roles in gene expression, cell division and development and control of immunological and reproductive functions, iron and zinc are important elements for human health. However, the investigation of such elements in various genotypes of rice is still unknown. In our investigation, high concentrations of Fe and Zn mineral nutrients were found among these genotypes M.J 5460S/SK105-1, M.J 5460S/GZ7768-1, M.J 5460S/G177-1, M.J 5460S/SK105-3 and M.J 5460S/SK106-4. Improved Japonica rice lines could be developed for higher yield and quality under Egyptian conditions, contributing to an increase in total production and export power. Zinc and iron deficiency will increase the chance and severity of common childhood infections, ultimately moving the survival of babies, and contributes to reduced growth of young kids. The latest estimates indicate that regarding 4% of death rate and disability-adjusted life-years worldwide may be answerable for zinc and iron deficiency. Additionally, such components square measure terribly important to support the system of human immunity within the fight against pandemic diseases such as Covid-19. To avoid Zn and Fe deficiency in rice as one of the most reliable food crops, measures are urgently needed for these reasons. However, more research is needed to investigate the potential impacts of climate change on such improved genotypes in order to innovate the appropriate adaptation options required for sustainable crop production.

## Supplemental Information

10.7717/peerj.11592/supp-1 Supplemental Information 1 Raw dataset for all genotypes and treatments. Three replications for the studied traits per sheet.

10.7717/peerj.11592/supp-2 Supplemental Information 2 Rice parental lines, parentage origin and grain type.

## Additional Information and Declarations

### Data Availability

The following information was supplied regarding data availability: The raw measurements are available in the Supplemental File.
