# Supplementary material for: Developing new lines of Japonica rice for higher quality and yield under arid conditions
Source: PeerJ. 2021 Jun 14;9:e11592. doi: 10.7717/peerj.11592 (PMC8210806; doi:10.7717/peerj.11592)
Supplement: Supplemental Information 2 [file peerj-09-11592-s002.docx]

**Table S1** Rice parental lines, parentage origin and grain type

| No. | Entries | Parentage | Origin | Grain type |
| --- | --- | --- | --- | --- |
| 1 | M.J 5460S | rT60-6 MS | China | Short |
| 2 | Giza 177 | [Giza 171] Ymji No.1 // PiNo.4 | Egypt | Short |
| 3 | Sakha105 | (GZ 5581 x GZ 4316) | Egypt | Short |
| 4 | Sakha106 | (Giza 177 x Hexi 30) | Egypt | Short |
| 5 | GZ.7768 | (GZ.5320 x Taninung 70) | Egypt | Short |
|  |  |  |  |  |

**Table S2** Mean performance of genotypes for yield and its components of Japonica and Indica varieties.

| No | Genotypes  (F_n_) | Days to heading | Plant height  (cm) | Panicle  plant^-1^ | Panicle weight  (g) | Panicle length  (cm) | Total grain panicle ^-1^ | Yield m^-2^  (kg) | Harvest index (%) |
| --- | --- | --- | --- | --- | --- | --- | --- | --- | --- |
|  |  |  |  |  |  |  |  |  |  |
| 1 | M.J 5460S/G177-1 | 89.6±0.6 | 84.0±2.5 | 16.7±0.4 | 5.8±0.1 | 20.3±0.1 | 245.7±5 | 1.4±0.05 | 39.9±0.3 |
| 2 | M.J 5460S/G177-2 | 90.5±0.7 | 101.0±3.0 | 18.7±0.4 | 4.5±0.1 | 20.0±0.1 | 196.7±4 | 1.2±0.06 | 43.6±0.1 |
| 3 | M.J 5460S/G177-3 | 90.5±0.7 | 87.0±2.8 | 15.7±0.5 | 7.5±0.1 | 20.3±0.1 | 264.7±3 | 1.3±0.06 | 37.3±0.2 |
| 4 | M.J 5460S/G177-4 | 90.3±0.7 | 82.0±3.5 | 14.3±0.3 | 6.3±0.1 | 19.4±0.1 | 215.7±4 | 1.5±0.04 | 49.1±0.1 |
| 5 | M.J 5460S/G177-5 | 90.4±0.8 | 77.0±3.0 | 15.3±0.5 | 6.5±0.1 | 19.4±0.1 | 197.7±6 | 1.2±0.06 | 41.3±0.4 |
| 6 | M.J 5460S/G177-6 | 96.4±0.9 | 107.0±4.0 | 22.3±0.2 | 3.5±0.2 | 20.7±0.1 | 166.7±8 | 1.2±0.06 | 49.5±0.1 |
| 7 | M.J 5460S/G177-7 | 99.9±0.9 | 110.0±4.2 | 21.3±0.3 | 5.9±0.2 | 22.1±0.2 | 213.7±4 | 1.3±0.07 | 43.3±0.4 |
| 8 | M.J 5460S/G177-8 | 99.9±0.5 | 108.0±4.2 | 21.7±0.3 | 3.9±0.2 | 20.8±0.2 | 174.7±5 | 1.1±0.07 | 41.9±0.2 |
| 9 | M.J 5460S/G177-9 | 99.0±0.7 | 122.0±4.0 | 24.3±0.3 | 4.7±0.1 | 20.4±0.2 | 144.7±9 | 1.2±0.08 | 42.8±0.3 |
| 10 | M.J 5460S/G177-10 | 99.4±0.8 | 124.0±4.2 | 23.3±0.2 | 4.5±0.2 | 19.6±0.2 | 184.7±7 | 1.2±0.09 | 49.9±0.1 |
| 11 | M.J 5460S/ SK105-1 | 95.2±0.9 | 87.0±3.5 | 16.3±0.3 | 5.6±0.2 | 17.8±0.1 | 194.7±7 | 1.2±0.09 | 40.0±0.1 |
| 12 | M.J 5460S/ SK105-2 | 95.6±1.0 | 117.0±3.2 | 30.0±0.2 | 6.4±0.1 | 22.2±0.1 | 238.7±8 | 1.1±0.08 | 42.4±0.2 |
| 13 | M.J 5460S/ SK105-3 | 92.5±0.9 | 107.0±3.0 | 14.3±0.5 | 5.7±0.1 | 21.1±0.2 | 185.7±9 | 1.2±0.09 | 42.3±0.2 |
| 14 | M.J 5460S/Sk106-1 | 98.2±0.5 | 117.0±3.5 | 16.7±0.4 | 6.9±0.1 | 21.1±0.3 | 207.7±8 | 1.4±0.08 | 43.8±0.2 |
| 15 | M.J 5460S/Sk106-2 | 96.0±0.6 | 90.0±3.2 | 16.7±0.4 | 6.2±0.1 | 23.1±0.1 | 211.7±5 | 1.3±0.09 | 42.2±0.1 |
| 16 | M.J 5460S/Sk106-3 | 105.2±0.7 | 76.0±3.4 | 19.0±0.3 | 6.5±0.1 | 22.1±0.2 | 209.7±4 | 1.3±0.08 | 43.7±0.2 |
| 17 | M.J 5460S/Sk106-4 | 96.2±0.8 | 110.0±4.5 | 20.7±0.2 | 6.1±0.1 | 22.2±0.2 | 212.7±5 | 1.5±0.07 | 49.8±0.3 |
| 18 | M.J 5460S/Sk106-5 | 105.2±0.9 | 107.0±4.4 | 27.3±0.2 | 5.5±0.1 | 22.4±0.2 | 187.7±6 | 1.3±0.07 | 34.7±0.5 |
| 19 | M.J 5460S/Sk106-6 | 98.2±0.8 | 114.0±4.0 | 21.7±0.3 | 6.0±0.1 | 24.2±0.2 | 251.7±5 | 1.5±0.06 | 42.1±0.6 |
| 20 | M.J 5460S/Sk106-7 | 97.2±0.9 | 117.0±5.2 | 22.3±0.4 | 6.3±0.1 | 20.5±0.2 | 180.7±8 | 1.4±0.05 | 45.1±0.4 |
| 21 | M.J 5460S/Sk106-8 | 100.2±0.9 | 110.0±5.0 | 27.0±0.1 | 4.3±0.1 | 21.1±0.2 | 200.7±6 | 1.2±0.05 | 38.3±0.7 |
| 22 | M.J 5460S/Sk106-9 | 99.8±0.8 | 107.0±4.5 | 22.3±0.2 | 4.7±0.2 | 20.4±0.2 | 186.7±9 | 1.2±0.05 | 40.8±0.6 |
| 23 | M.J 5460S/Sk106-10 | 101.5±0.7 | 122.0±4.2 | 27.3±0.2 | 7.0±0.2 | 22.4±0.1 | 227.7±9 | 1.2±0.04 | 37.1±0.5 |
| 24 | M.J 5460S/SK106-11 | 103.8±0.8 | 120.0±3.0 | 18.7±0.4 | 4.9±0.3 | 19.3±0.1 | 208.7±8 | 1.0±0.03 | 39.5±0.5 |
| 25 | M.J 5460S/GZ7768-1 | 98.5±0.9 | 82.0±3.5 | 24.3±0.1 | 6.2±0.1 | 20.3±0.2 | 227.7±7 | 1.5±0.02 | 49.8±0.5 |
| 26 | M.J 5460S/GZ7768-2 | 96.8±1.0 | 86.7±3.2 | 24.3±0.1 | 4.5±0.2 | 19.1±0.3 | 240.7±6 | 1.2±0.05 | 43.1±0.6 |
| 27 | M.J 5460S/GZ7768-3 | 99.5±1.1 | 93.3±5.5 | 22.3±0.1 | 4.8±0.2 | 23.1±0.3 | 187.7±5 | 1.2±0.07 | 49.9±0.7 |
| 28 | M.J 5460S/GZ7768-4 | 99.8±1.2 | 96.3±5.0 | 23.7±0.2 | 6.2±0.1 | 24.3±0.1 | 266.7±6 | 1.4±0.02 | 44.8±0.8 |
| 29 | M.J 5460S/GZ7768-5 | 101.5±1.3 | 93.3±4.5 | 24.3±0.2 | 4.9±0.2 | 24.6±0.2 | 170.7±9 | 1.4±0.02 | 44.4±0.5 |
| 30 | M.J 5460S/GZ7768-6 | 102.5±1.3 | 114.0±3.2 | 23.7±0.2 | 5.9±0.2 | 23.1±0.2 | 223.7±9 | 1.4±0.03 | 35.1±0.5 |
| 31 | M.J 5460S/GZ7768-7 | 101.1±0.9 | 122.0±3.5 | 25.3±0.2 | 6.0±0.2 | 20.2±0.2 | 208.7±8 | 1.4±0.04 | 49.8±0.3 |
| 32 | M.J 5460S/GZ7768-8 | 97.8±0.9 | 92.0±4.8 | 24.7±0.3 | 6.1±0.1 | 21.4±0.2 | 248.7±9 | 1.5±0.04 | 42.5±0.2 |
| 33 | M.J 5460S/GZ7768-9 | 98.5±0.9 | 92.0±5.0 | 24.7±0.5 | 6.3±0.1 | 22.0±0.2 | 228.7±9 | 1.4±0.04 | 42.1±0.3 |
| 34 | M.J 5460S/GZ7768-10 | 103.5±0.8 | 110.0±3.5 | 19.0±0.5 | 6.9±0.1 | 21.8±0.1 | 297.7±3 | 1.4±0.03 | 46.0±0.4 |
| 35 | Giza 178 (CK) | 101.0±0.7 | 96.3±3.2 | 22.7±0.3 | 4.5±0.1 | 23.0±0.1 | 187.0±5 | 1.1±0.02 | 48.6±0.4 |
| 36 | Giza 177(CK) | 92.0±0.6 | 97.7±3.5 | 19.0±0.6 | 4.0±0.2 | 21.2±0.1 | 158.0±9 | 1.0±0.02 | 40.4±0.4 |
| 37 | Giza 179(CK) | 92.0±0.5 | 84.3±2.8 | 24.7±0.3 | 4.1±0.3 | 22.5±0.1 | 210.0±8 | 1.1±0.01 | 43.6±0.5 |
| 38 | Sakha Super 300(CK) | 109.0±0.6 | 113.3±2.9 | 20.3±0.4 | 5.3±0.2 | 22.3±0.1 | 235.0±8 | 1.2±0.03 | 43.7±0.5 |
| 39 | Sakha 101(CK) | 108.0±0.7 | 90.0±3.0 | 21.7±0.3 | 4.3±0.2 | 24.7±0.1 | 172.0±9 | 1.1±0.09 | 46.0±0.6 |
| 40 | Sakha 108(CK) | 105.0±0.8 | 87.3±3.5 | 24.7±0.3 | 3.6±0.2 | 20.8±0.2 | 182.0±9 | 1.2±0.08 | 44.8±0.9 |
|  | Mean | 98.4 | 101.3 | 21.6 | 5.5 | 21.4 | 208.8 | 1.3 | 43.5 |

Values = mean± Sdv

**Table S3** Mean performance of different genotypes for grain quality traits.

| No | Genotypes  (F_n_) | Hulling  % | Milling  % | Head Rice % | Amylose  % | GT | Elongation | Paddy  grain shape | White  grain shape |
| --- | --- | --- | --- | --- | --- | --- | --- | --- | --- |
| 1 | M.J 5460S/G177-1 | 80.3±0.7 | 73.8±0.5 | 70.3±1.5 | 18.0±0.8 | 7.0±0.8 | 32.3±4.0 | 2.2±0.1 | 1.8±0.1 |
| 2 | M.J 5460S/G177-2 | 79.0±0.8 | 70.2±0.6 | 65.3±1.2 | 20.2±0.7 | 5.0±0.9 | 17.2±3.5 | 2.5±0.1 | 2.1±0.3 |
| 3 | M.J 5460S/G177-3 | 81.4±0.5 | 73.8±0.7 | 70.0±1.6 | 19.9±0.6 | 5.0±0.9 | 35.0±2.5 | 2.4±0.1 | 1.9±0.2 |
| 4 | M.J 5460S/G177-4 | 79.0±0.6 | 70.2±0.8 | 66.7±1.7 | 20.6±0.7 | 5.0±0.8 | 41.0±2.2 | 2.2±0.2 | 1.8±0.1 |
| 5 | M.J 5460S/G177-5 | 83.7±0.7 | 73.3±0.7 | 66.7±1.2 | 18.7±0.8 | 6.0±0.7 | 28.2±3.2 | 2.3±0.2 | 1.9±0.3 |
| 6 | M.J 5460S/G177-6 | 83.7±0.5 | 73.8±0.5 | 70.0±1.4 | 18.7±0.8 | 6.2±0.6 | 17.1±2.8 | 2.3±0.2 | 2.1±0.1 |
| 7 | M.J 5460S/G177-7 | 83.7±0.5 | 71.2±0.6 | 69.1±1.5 | 19.8±0.7 | 5.0±0.5 | 19.2±2.5 | 2.6±0.1 | 2.1±0.1 |
| 8 | M.J 5460S/G177-8 | 81.4±0.7 | 72.5±0.5 | 70.0±1.7 | 17.1±0.6 | 6.0±0.4 | 18.0±3.5 | 2.3±0.1 | 1.7±0.2 |
| 9 | M.J 5460S/G177-9 | 81.4±0.6 | 74.8±0.4 | 70.0±2.0 | 18.2±0.7 | 5.0±0.5 | 17.6±3.2 | 2.3±0.2 | 2.0±0.2 |
| 10 | M.J 5460S/G177-10 | 88.8±0.5 | 74.4±0.5 | 60.0±2.1 | 20.1±0.7 | 6.0±0.7 | 37.8±3.1 | 2.3±0.2 | 1.9±0.1 |
| 11 | M.J 5460S/ SK105-1 | 83.7±0.4 | 74.8±0.7 | 71.2±2.3 | 19.3±0.8 | 7.0±0.8 | 17.0±4.0 | 2.3±0.2 | 1.9±0.1 |
| 12 | M.J 5460S/ SK105-2 | 79.0±0.5 | 70.2±0.5 | 64.0±2.1 | 19.5±0.9 | 4.0±0.9 | 18.9±2.2 | 2.5±0.1 | 2.0±0.2 |
| 13 | M.J 5460S/ SK105-3 | 82.8±0.5 | 73.2±0.6 | 70.6±2.3 | 19.4±0.7 | 7.0±0.8 | 27.1±2.5 | 2.3±0.1 | 2.0±0.1 |
| 14 | M.J 5460S/Sk106-1 | 80.6±0.5 | 72.2±0.5 | 70.0±2.3 | 18.4±0.6 | 7.0±0.7 | 35.4±3.5 | 2.3±0.1 | 2.0±0.2 |
| 15 | M.J 5460S/Sk106-2 | 80.4±0.4 | 74.8±0.5 | 56.7±2.1 | 18.7±0.7 | 6.0±0.5 | 24.8±3.8 | 2.6±0.1 | 2.1±0.1 |
| 16 | M.J 5460S/Sk106-3 | 80.4±0.3 | 74.8±0.5 | 66.7±2.2 | 19.0±0.6 | 6.0±0.7 | 39.9±4.5 | 2.4±0.2 | 2.0±0.2 |
| 17 | M.J 5460S/Sk106-4 | 83.7±0.3 | 74.8±0.8 | 66.7±2.0 | 18.1±0.7 | 6.0±0.8 | 20.1±2.8 | 2.3±0.2 | 2.0±0.2 |
| 18 | M.J 5460S/Sk106-5 | 80.4±0.2 | 73.5±0.7 | 51.6±2.5 | 19.1±0.6 | 6.0±0.7 | 32.2±3.5 | 2.7±0.2 | 2.1±0.1 |
| 19 | M.J 5460S/Sk106-6 | 83.7±0.5 | 71.5±0.9 | 69.0±2.1 | 18.3±0.6 | 6.0±0.6 | 28.8±3.8 | 2.3±0.2 | 2.2±0.1 |
| 20 | M.J 5460S/Sk106-7 | 74.1±0.5 | 70.2±0.8 | 66.7±2.3 | 18.9±0.7 | 5.0±0.4 | 20.9±4.5 | 2.6±0.1 | 2.1±0.3 |
| 21 | M.J 5460S/Sk106-8 | 79.3±0.6 | 69.8±0.9 | 63.8±2.4 | 18.8±0.6 | 5.0±0.7 | 26.7±4.2 | 2.4±0.1 | 2.0±0.1 |
| 22 | M.J 5460S/Sk106-9 | 79.7±0.7 | 66.8±0.8 | 63.6±2.2 | 18.7±0.7 | 6.0±0.8 | 31.7±4.1 | 2.4±0.1 | 2.1±0.1 |
| 23 | M.J 5460S/Sk106-10 | 83.4±0.5 | 73.5±0.5 | 70.0±2.1 | 18.8±0.6 | 5.0±0.7 | 26.7±3.5 | 2.2±0.2 | 2.2±0.1 |
| 24 | M.J 5460S/SK106-11 | 79.6±0.8 | 74.4±0.4 | 71.3±2.2 | 20.9±0.7 | 6.0±0.7 | 21.3±3.1 | 2.2±0.2 | 2.1±0.1 |
| 25 | M.J 5460S/GZ7768-1 | 80.4±0.9 | 72.8±0.5 | 70.3±2.2 | 19.3±0.5 | 6.0±0.6 | 30.4±2.8 | 2.4±0.2 | 1.7±0.1 |
| 26 | M.J 5460S/GZ7768-2 | 83.7±0.5 | 73.2±0.6 | 70.3±2.3 | 20.8±0.7 | 6.0±0.5 | 30.2±3.5 | 2.5±0.1 | 2.2±0.2 |
| 27 | M.J 5460S/GZ7768-3 | 81.6±0.5 | 66.8±0.9 | 63.3±2.4 | 18.9±0.7 | 5.1±0.4 | 35.2±3.8 | 2.2±0.2 | 2.0±0.2 |
| 28 | M.J 5460S/GZ7768-4 | 80.4±0.6 | 74.8±0.7 | 71.3±2.6 | 18.7±0.8 | 6.0±0.5 | 14.2±2.8 | 2.3±0.1 | 2.1±0.2 |
| 29 | M.J 5460S/GZ7768-5 | 83.7±0.7 | 73.5±0.6 | 66.7±3.2 | 20.1±0.7 | 5.0±0.5 | 36.7±3.8 | 2.2±0.2 | 1.9±0.1 |
| 30 | M.J 5460S/GZ7768-6 | 82.0±0.6 | 69.2±0.5 | 66.0±3.1 | 20.9±0.8 | 6.0±0.4 | 36.8±4.5 | 2.4±0.1 | 2.1±0.2 |
| 31 | M.J 5460S/GZ7768-7 | 78.0±0.7 | 60.2±0.6 | 56.7±3.5 | 19.9±0.7 | 6.0±0.3 | 27.4±4.2 | 2.4±0.2 | 2.0±0.1 |
| 32 | M.J 5460S/GZ7768-8 | 80.4±0.5 | 73.5±0.7 | 63.3±3.4 | 20.6±0.6 | 7.0±0.5 | 40.0±4.5 | 2.2±0.1 | 2.0±0.2 |
| 33 | M.J 5460S/GZ7768-9 | 80.4±0.4 | 70.2±0.8 | 63.3±3.5 | 19.7±0.7 | 7.0±0.4 | 17.8±4.3 | 2.8±0.1 | 1.9±0.5 |
| 34 | M.J 5460S/GZ7768-10 | 80.4±0.5 | 70.2±0.8 | 60.0±3.1 | 18.3±0.8 | 6.0±0.5 | 25.5±5.5 | 2.2±0.2 | 2.4±0.1 |
| 35 | Giza 178 (CK) | 80.4±0.5 | 70.5±0.9 | 66.3±2.2 | 20.2±0.7 | 7.0±0.6 | 28.4±5.1 | 2.5±0.1 | 2.0±0.2 |
| 36 | Giza 177(CK) | 80.7±0.6 | 73.2±0.8 | 69.7±2.1 | 18.4±0.8 | 7.0±0.7 | 38.5±4.5 | 2.3±0.1 | 1.8±0.1 |
| 37 | Giza 179(CK) | 82.2±0.5 | 71.8±0.8 | 62.3±2.2 | 19.5±0.9 | 6.0±0.8 | 33.3±3.5 | 2.3±0.1 | 1.8±0.1 |
| 38 | Sakha Super 300(CK) | 83.7±0.4 | 73.2±0.7 | 66.7±2.3 | 18.8±0.8 | 7.0±0.8 | 44.5±3.2 | 2.1±0.1 | 1.7±0.2 |
| 39 | Sakha 101(CK) | 80.2±0.7 | 71.5±0.6 | 65.3±2.1 | 18.0±0.8 | 7.0±0.6 | 38.0±3.1 | 2.4±0.1 | 1.7±0.3 |
| 40 | Sakha 108(CK) | 80.1±0.5 | 71.7±0.5 | 64.7±2.3 | 18.3±0.8 | 7.0±0.5 | 40.5±2.8 | 2.2±0.1 | 1.8±0.2 |
|  | Mean | 81.28 | 71.96 | 66.44 | 19.19 | 5.98 | 28.80 | 2.35 | 1.98 |

GT: Gelatinization temperature, values = means ±Stdev

**Table S4** Correlation co-efficient among yield and it is components and grain quality traits.

|  | Milling % | Head Rice % | Amylose % | GT | Elongation | Paddy grain shape | White grain shape | Days to heading | Plant height | Panicle plant | Panicle weight | Panicle Length | Total grain panicle | **Yield kg m ^-2^** | Harvest index |
| --- | --- | --- | --- | --- | --- | --- | --- | --- | --- | --- | --- | --- | --- | --- | --- |
| Hulling % | 0.424  ** | 0.170  NS | 0.036  NS | 0.140  NS | 0.122  NS | -0.269  * | -0.03  NS | 0.001  NS | 0.040  NS | -0.06  NS | -0.15  NS | 0.023  NS | -0.13  NS | -0.08 NS | -0.03  NS |
| Milling % |  | 0.336* | -0.152  NS | 0.171  NS | -0.05  NS | -0.183  NS | -0.07  NS | -0.101  NS | -0.22  * | -0.25  * | 0.028  NS | -0.026  NS | -0.02  NS | -0.10  NS | 0.001  NS |
| Head Rice % |  |  | -0.071  NS | 0.052  NS | -0.19  NS | -0.300  ** | -0.08  NS | -0.243  * | -0.06  NS | -0.32  * | -0.01  NS | -0.203  NS | -0.11  NS | -0.11  NS | -0.01  NS |
| Amylose % |  |  |  | -0.19  NS | 0.137  NS | 0.159  NS | 0.175  NS | -0.105  NS | -0.05  NS | 0.078 | 0.087  NS | -0.201  NS | 0.051  NS | -0.02  NS | 0.169  NS |
| GT |  |  |  |  | 0.279  * | -0.113  NS | -0.28  * | 0.156  NS | -0.24  * | -0.29  ** | -0.17  NS | -0.018  NS | -0.01  NS | -0.02  NS | -0.03  NS |
| Elongation |  |  |  |  |  | -0.360  ** | -0.39  ** | 0.205  NS | -0.29  * | -0.10 | -0.03  NS | 0.136  NS | 0.069  NS | 0.114  NS | 0.008  NS |
| Paddy grain shape |  |  |  |  |  |  | 0.236  * | -0.002  NS | -0.04  NS | 0.244* | 0.120  NS | 0.072  NS | 0.042  NS | 0.025  NS | -0.06  NS |
| White grain shape |  |  |  |  |  |  |  | 0.086  NS | 0.399  ** | 0.399** | 0.229  * | 0.111  NS | 0.148  NS | 0.157  NS | 0.280  * |
| Days to heading |  |  |  |  |  |  |  |  | 0.313  * | 0.441** | -0.15  NS | 0.416  ** | -0.04  NS | 0.093  NS | -0.05  NS |
| Plant height |  |  |  |  |  |  |  |  |  | 0.332  * | -0.03  NS | 0.044 | -0.06  NS | 0.067  NS | -0.10  NS |
| panicle plant |  |  |  |  |  |  |  |  |  |  | -0.24  * | 0.331  * | -0.04  NS | 0.050  NS | 0.651  ** |
| Panicle weight |  |  |  |  |  |  |  |  |  |  |  | 0.056 | 0.194  NS | 0.246  * | 0.112  NS |
| Panicle length |  |  |  |  |  |  |  |  |  |  |  |  | 0.194  NS | 0.246  * | 0.112  NS |
| Total grain panicle |  |  |  |  |  |  |  |  |  |  |  |  |  | 0.814  ** | 0.513  ** |
| Yield kg m^-2^ |  |  |  |  |  |  |  |  |  |  |  |  |  |  | 0.462  ** |

n.s correlation is not significant * correlation is significant at the 0.05 level * * correlation is high significant at the 0.01 level
